# *Candida* biome of severe early childhood caries (S-ECC) and its cariogenic virulence traits

**DOI:** 10.1080/20002297.2020.1724484

**Published:** 2020-02-05

**Authors:** Kausar Sadia Fakhruddin, Lakshman Perera Samaranayake, Hiroshi Egusa, Hien Chi Ngo, Chamila Panduwawala, Thenmozhi Venkatachalam, Allagappan Kumarappan, Siripen Pesee

**Affiliations:** a Department of Preventive and Restorative Dentistry, University of Sharjah, Sharjah, UAE; b Faculty of Dentistry, The University of Hong Kong, Hong Kong Special Administrative Region, China; c Division of Molecular and Regenerative Prosthodontics, Tohoku University Graduate School of Dentistry, Sendai, Japan; d Research Institute of Medical and Health Sciences, University of Sharjah, Sharjah, UAE; e Faculty of Dentistry, Thammasat University, Pathum Thani, Thailand

**Keywords:** *Candida* species, severe early childhood caries (S-ECC), dentin caries, hydrolases, biofilm, calcium-release, acidogenicity, haemolysin, phopholipase, protease

## Abstract

The protected niche of deep-caries lesions is a distinctive ecosystem. We assessed the *Candida* biome and its cariogenic traits from dentin samples of 50 children with severe-early childhood caries (S-ECC). Asymptomatic, primary molars belonging to International Caries Detection and Assessment-ICDAS caries-code 5 and 6 were analyzed, and *C. albicans* (10-isolates), *C. tropicalis* (10), *C. krusei* (10), and *C. glabrata* (5) isolated from the lesions were then evaluated for their biofilm formation, acidogenicity, and the production of secreted hydrolases: hemolysins, phospholipase, proteinase and DNase. *Candida* were isolated from 14/43 ICDAS-5 lesions (32.5%) and 44/57 ICDAS-6 lesions (77.2%). Compared to, ICDAS-5, a significantly higher frequency of multi-species infestation was observed in ICDAS-6 lesions (p=0.001). All four candidal species (above) showed prolific biofilm growth, and an equal potency for tooth demineralization. A significant interspecies difference in the mean phospholipase, as well as proteinase activity was noted (p < 0.05), with *C. albicans* being the predominant hydrolase producer. Further, a positive correlation between phospholipase and proteinase activity of *Candida*-isolates was noted (r = 0.818, p < 0.001). Our data suggest that candidal mycobiota with their potent cariogenic traits may significantly contribute to the development and progression of S-ECC.

## Introduction

Early childhood caries (ECC) is the most ubiquitous, plaque biofilm-mediated, aggressive form of dental caries affecting children the world over [1,2]. A hypervirulent variant of this intractable disease is called severe-early childhood caries (S-ECC) [3]. According to International Caries Detection and Assessment System (ICDAS), S-ECC could sub-categorized as caries code-5, a distinct cavity with visible dentin, and caries code 6, an extensive caries lesion involving half/more than half of tooth. Code 5 and 6 are the most severe caries lesions as per the ICDAS classification system [4,5].

S-ECC is particularly rampant in the developing world due to the relatively wide and cheap availability and accessibility of sucrose substrates, and inadequate dental health care delivery systems [6]. Without appropriate intervention, S-ECC lesions may further progress, leading to extensive cavitation, reaching the pulp chamber, and compromising the longevity of the tooth, while simultaneously serving as a potent reservoir for systemically seeded infections [3,7,8].

The protected niche of deep cavitated lesions in S-ECC is a unique ecosystem. First, due to the relatively extreme depth, the carious lesion is poorly accessible to routine oral hygiene measures, as well as the salivary flushing mechanisms compounding the plaque biofilm accumulation. Second, the sugar pulses from sucrose-rich food frequently snacked by these children are retained over a prolonged period in such niches, providing a constant and a ready source of food supply for the resident microbiota. Both these conditions inevitably lead to a very low pH acidic locale, leading to the emergence, growth, and sustenance of a profuse biofilm, particularly rich in aciduric, and acidophilic microbiota [9].

It is generally recognized that the *mutans*-group streptococci are the prime movers of the caries process due to their acidogenic and aciduric nature [10]. However, several studies have now shown that the aciduric oral yeasts, mainly belonging to *Candida* species, frequently co-inhabit these lesions with *mutans*- streptococci, and significantly contribute to the caries process [11–14]. There is also a substantial body of data to indicate cross-kingdom synergistic, interactions between this fungus and cariogenic bacteria within such polymicrobial-biofilm habitats [15,16] making the eukaryote a candidate caries pathogen. Indeed, some have hypothesized that *Candida* species are secondary movers of the pathological process in deep carious lesions, primarily initiated by *mutans*-group streptococci [17]. In a recent, ultrastructural study Dige and Nyvad [18] have elegantly demonstrated the co-colonization of *Candida* species with streptococci in intact *in vivo* biofilms from carious lesions, and called for a detailed examination of diverse virulence attributes of the yeast species that modulate this ecosystem [18].

On closer examination, it is apparent that *Candida* species possess a slew of virulence traits that may contribute to dental decay [17,19–21]. First, these fungi are richly endowed with metabolic machinery required for dietary carbohydrate metabolism and the production of short-chain carboxylic acids such as acetates, and lactates, and contribute to the generation of an acidic milieu, which in turn facilitates the demineralization of dentinal tissues. The seminal work of Samaranayake et al, in early eighties, clearly demonstrated the acidogenic potential as well as the survival potential of various *Candida* species under such extremely adverse, low pH conditions [22,23]. Second, the yeasts possess critical attributes essential for synergistic biofilm development with cariogenic flora [15,24], including their ability to adhere avidly to abiotic surfaces and develop profuse biofilms whilst serving as anchor organisms providing a skeletal framework for the biofilm [2]. Several studies have reported the ability of *Candida albicans* in particular to produce extracellular hydrolases such as hemolysins, phospholipases, acidic hydrolases and DNases [20,22,25–27] which could contribute to the breakdown the organic structural components of the human dentin.

The oral mycobiome and the prevalence of it predominate constituent *Candida*, in childhood caries has been sparsely studied. In a recent preliminary study of 15 Australian children, Fechney et al. concluded that their oral mycobiome comprised at least 46 fungal species [14]. Further, they noted that the diversity of fungi was similar irrespective of the caries status of this small pediatric cohort, and caries influenced the abundance of specific fungi. However, as far as we are aware, no studies, to date have been performed to assess the prevalence of *Candida* species in S-ECC, and to evaluate their cariogenic traits that may contribute to the pathogenesis of the disease. Hence, the aim of this study was first, to characterize the prevalence of *Candida* species in S-ECC in a Middle East child cohort, with ICDAS caries code 5 and 6, and then to evaluate the biofilm formation, acidogenicity, and the production of four secreted hydrolases: hemolysin, phospholipases, proteinase, and DNase in a select group of 35 wild-type *C. albicans* (10 strains), *C tropicalis* [10], *C. krusei* [10] and *C. glabrata* [5] isolated from such lesions.

## Materials and methods

### Study subjects

As per protocol approved by the Research Ethics Committee, University of Sharjah (REC-18-02-18-03), 50 children, aged-48-months to 72-months, attending a regular teaching clinic at the University Dental Hospital Sharjah, UAE, were invited to participate in the study. After obtaining informed consent from the parents of each child participant, a full dental examination was carried out for all healthy, cooperative participants. Children with more than five-decayed teeth and having at least two asymptomatic primary molars with occlusal or proximal carious lesions involved were selected by a calibrated examiner (KSF). The severity of cavitated lesions was determined by the examiner according to ICDAS classification; viz. code 5 being a distinct cavity with visible dentine involving less than half the tooth surface, and code 6, an extensive and distinct cavity with visible dentine affecting more than half of the surface.

The exclusion criteria were children on antibiotics over the last 4-weeks before sample collection, those wearing orthodontic appliance/s or with congenital tooth anomaly, or with any likelihood of pulp exposure during the caries excavation process. Further, dentine samples from endodontically treated teeth, or when gingival bleeding contaminated the cavity during the sample collection process were also excluded.

### Caries diagnosis

Caries status was recorded using the WHO criterion of decayed, missing, and filled (dmft) tooth index. The severity of cavitated lesions was ascertained as either caries-code 5 or 6 according to ICDAS- caries criteria [4]. One trained pediatric dentist conducted the clinical examination and sample collection throughout the studies (KSF).

### Sample collection

A total of 100 infected-dentine samples from 50 children (two samples each) were aseptically collected by a single trained collector (KSF) from both occlusal and proximal, symptom-free, caries active, deep-dentin lesions belonging to ICDAS caries-code 5 and code 6.

Samples were collected using a sterile spoon excavator after cleaning and drying the cavities with a prophy brush, without using prophy paste. Each sample was split in two and one aliquot was placed in an Eppendorf centrifuge tube (1.5 ml) containing 300 µl of Phosphate buffered saline (PBS) for multiplex PCR, and the second aliquot in Brain Heart Infusion (BHI) Broth (Thermo Scientific Remel, USA), for culture, and immediately frozen at −20°C until further use.

In the laboratory, one aliquot in BHI broth was cultured aerobically on Sabouraud dextrose agar (SDA) at 37°C for 48 hr, and the resultant growth observed. All samples which yielded yeast growth were then sub-cultured on CHROMagar (HiCrome™ Candida Differential Agar, M1297A) for 24 hr. afterwards, pure cultures of different species were obtained by selecting colony forming units on the basis of their colonial appearance on CHROM agar. The different candidal species thus obtained from each sample was then sub cultured in Sabouraud dextrose broth for 24 hr. to evaluate the virulence attributes.

### DNA isolation and multiplex PCR

The second aliquot in PBS, from yeast positive clinical samples was then subjected to multiplex PCR amplification method of Trost et al [28], with minor modifications The multiplex PCR was based on the amplification of two fragments from the ITS1 and ITS2 regions by the combination of two-yeast-specific and six-species-specific primers in a single PCR reaction [28] and our method permitted the identification of up to six clinically relevant yeasts of the *Candida* genus that were found in our clinical samples, namely *C. albicans, C. glabrata, C. parapsilosis, C. tropicalis, C. krusei*, and *C. dubliniensis*. (Table 1).

**Table 1.** Amplicon sizes (base pairs) results from multiplex PCR amplification using yeast specific (Universal-UNI1 and UNI2) and corresponding species-specific primers of *Candida* spp

Species	Primer	Sequence (5ʹ-3ʹ)	Amplicon size (bp)
	UNI 1 UNI 2	GTCAAACTTGGTCATTTA TTCTTTTCCTCCGCTTATTG	
*C. albicans*	Calb	AGCTGCCGCCAGAGGTCTAA	583/446
*C. tropicalis*	Ctro	GATTTGCTTAATTGCCCCAC	583/507
*C. krusei*	Ckru	CTGGCCGAGCGAACTAGACT	590/169
*C. glabrata*	Cgla	TTGTCTGAGCTCGGAGAGAG	929/839
*C. dubliniensis*	Cdub	CTCAAACCCCTAGGGTTTGG	591/217
*C. parapsilosis*	Cpar	GTCAACCGATTATTTAATAG	570/370

DNA extraction of the collected infected-dentine samples was performed using MasterPure™ Complete DNA and RNA Purification (Epicenter, USA), following the manufacturer’s guidelines. The quality and the quantity of the extracted DNA were assessed using a Colibri Microvolume Spectrometer (Titertek-Berthold Detection Systems GmbH, Germany). DNA samples were considered pure if the A260/280 ratio were more than 1.8, and A260/230 values were in the range of (1–2.2).

PCR was performed under the following cycling conditions: 40 cycles of 15 secs at 94°C, then 30 secs at 55°C, and45 secs at 65°C, after a 10-minute initial period of DNA denaturation and enzyme activation at 94°C [29]. All PCR-reaction products were evaluated by electrophoresis in 2.0% (w/v) agarose gels run at 90 V for 60 mins. Identified poly-fungal samples were re-confirmed by quantitative PCR analysis using species-specific primers.

### Candida isolates

We restricted our investigations of the virulence attributes to randomly chosen 35 isolates belonging to four predominant candidal species *C. albicans* (10 strains), *C tropicalis* [10], *C. krusei* [10], and *C. glabrata* [5]. The phenotypes of the isolates, as identified by characteristic growth on CHROMagar (HiCrome™ Candida Differential Agar, M1297A) was reconfirmed by PCR identification prior to the virulence assays.

### Evaluation of biofilm formation

The method of Jin et al. [30], with modifications was used to develop candidal biofilms of the selected 35 yeast isolates belonging to four different candidal species, as follows. Flat-bottom 96-well microtiter plates (Corning, 3370 Polypropylene) were used for biofilm formation. Cell suspensions were further diluted to a final concentration of 10^3^ cells/ml in the RPMI-1640 medium w/L-glutamine, 0.2% glucose, and 0.165 moles/l MOPS buffer w/o sodium bicarbonate (AT180, RPMI-1640, Himedia). RPMI 1640 contains 0.2%D-glucose, but for the biofilm assay, we supplemented with D-glucose up to 2%, as a final concentration.

The plates were then incubated at 37°C for 24–48 hrs. in a shaker incubator (Thermo Scientific 4430) at 90 rpm. After biofilm formation at 24 h and 48 h, the medium was carefully aspirated using multichannel pipette without disrupting the biofilms. The plates were washed thrice with sterile PBS (200 µl/well) and were drained in an inverted position by blotting with a paper towel after the last wash, to remove any residual PBS.

The quantitation of biofilms was later performed by the XTT reduction assay. Before each test, a new XTT solution (Sigma-Aldrich) was prepared by reconstituting 4 mg of XTT in sterilized-filtered 10 ml PBS. This solution was added with menadione (Sigma-Aldrich) stock solution prepared in acetone. Using a multichannel pipette, 100 µl of XTT/menadione solution was added to each well containing pre-washed biofilm. The plates were then incubated for 2 hours at 37°C, after which 80 µl of the resultant colored supernatant from each well was then transferred to a new microtiter plate, and its absorbance was gauged at 490 nm using a spectrophotometer.

### Calcium-release assay for acidogenicity evaluation

To evaluate the acidogenicity of the 35 clinical isolates of *Candida* species, in terms of degrading the mineralized components of the tooth structure, a calcium-release assay was performed, according to Nikawa et al. [31] and Szabo et al. [32], with some modifications. Acidogenicity was concomitantly evaluated by pH measurements of the incubating media.

Briefly, dental root-discs obtained from two sound mesiodens and six sound premolars were sterilized using wet-heat under pressure and treated under UV radiation for 3 hours [33]. The teeth were sectioned and placed at the bottom of 12-culture multi-well plates (Corning® Costar® TC-Treated). The structural components of the sectioned teeth comprised mainly of dentine with a marginal layer of cementum.

The yeast suspensions belonging to four species were adjusted to an optical density (OD) of 1.0 at 530 nm (1x10^8^ cells/ml), and 50 µl of each of the selected isolates were inoculated into each well containing a dental disc. 950 µl of SDB containing 50 mM glucose (adjusted to pH 7.0 using NaOH) was then added to each well followed by incubation for 48, 96, and 144 h at 37°C. At the above time-points the pH level was assessed using a pH meter (Portable pH meter- H1991, Hanna, USA).

The release of calcium ion during degradation of mineralized tooth structure of the teeth was measured with calcium colorimetric assay kit (Abcam- Colorimetric, ab102505). The calcium ion concentration was determined by the chromogenic complex formed by calcium ion and *o*-cresolphthalein, which was proportional to the concentration of calcium ion. A total of 90 μL chromogenic reagent and 60 μL calcium assay buffer were added to each well of 96-well plate containing 50 μL of standards, samples, and controls. After mixing, the reaction system was incubated at room temperature for 5–10 mins, protected against light, before absorbance measurement at OD575 nm. The mean Ca++ release was obtained from readings taken on three independent occasions.

### Hemolysin assay

Hemolysin production of 35 *Candida* spp. was evaluated as described by Luo et al. [34] An inoculum size adjusted to 10^8^ cells/ml was prepared for each isolate, and 10 µl of each yeast suspension were spot inoculated on blood agar and the plates incubated for 48 h at 37°C. The variable expression of hemolysins by *Candida* species was viewed with transmitted light and assessed semi-quantitatively by the presence of a distinct-translucent halo around the inoculum site, indicating positive hemolytic activity.

### Phospholipase assay

*Candida* isolates were assayed for phospholipase activity on egg-yolk agar, according to the method described by Samaranayake et al. [35]. The egg-yolk medium comprising 13 g SDA, 0.11 g CaCl_2_, 11.7 g NaCl, and 10% egg-yolk emulsion was prepared. A10 µl of yeast cell suspensions, adjusted to 10^8^ cells/ml were spot inoculated on an egg-yolk agar and left to dry at room temperature. A 5 µl of saline was overlaid on the plate, and after drying at room temperature, each culture was incubated at 37°C for 48 h. Measurement of the zone of phospholipase activity (Pz) was conducted according to the method explained by Price et al. (1982). The diameter of the precipitation zone around the colony was established as the ratio of the diameter of the colony to the diameter of the colony and the precipitation zone expressed in mm [36].

### Proteinase assay

Proteinase enzyme activity of *Candida* spp. was performed in terms of bovine serum albumin (BSA) degradation as per the technique of Ruma-Haynes et al. with some modifications [37]. The BSA test medium consisted of 20 g of dextrose, 1 g K_2_HPO_4_, 0.5 g MgSO_4_, 0.2 g yeast extract, 15 g of agar, 2 g of bovine serum albumin. Briefly, an 18 h yeast cell suspension adjusted to 10^8^ cells was prepared, and the 10 µl suspension was inoculated onto the BSA plate and incubated at 37°C for five days. The plates were flooded with 1.25% Amido black stain (MB165-Amido black 10B) in 90% methanol and 10% acetic acid and allowed to stand for 10 minutes for the staining. After de-staining using 15% acetic acid for 20 minutes, plates were washed twice with PBS and allowed to dry at room temperature. Proteinase activity (Pr_z_) was determined as the ratio of the colony diameter to that of clear zone proteolysis expressed in mm.

### DNase assay

For the DNase assay, in brief, an 18-hr yeast cell suspension adjusted to 10^8^ cells were prepared, and the 10 µl suspension was inoculated onto DNase agar plates and incubated at 30°C for seven days as described by Sanchez and Colom [38]. The DNase results were expressed as either negative or positive depending on the absence or presence of a clear halo around the colony.

All assays were conducted on three separate occasions for each yeast-isolate tested.

## Statistical analysis

Numerical data obtained were analyzed using *t*-tests. Chi-square, Fischer exact tests, and analysis of variance (ANOVA) to compare the results between *Candida* species. The degree of correlation between the severity of caries lesion and *Candida* spp. Pearson correlation analysis was used. All results were considered significant at p ≤ 0.05.

## Results

Yeasts were detected in deep-dentine caries lesions of 37 of 50 (74%) children, (mean-age 5.2 (±0.82) years with S-ECC, enrolled in the study. Overall, the mean number of decayed teeth per child in our cohort was 8.38 (±2.86), compared to 8.59 (±2.89) decayed teeth in a sub-set of children who were yeast positive. Of 37 children with yeast-positive lesions evaluated (50-occlusal and 50-proximal lesions; total 100) 43; (43%) were ICDAS- 5 and 57 (57%) were ICDAS- 6.

In terms of the species distribution of *Candida* species, *C. krusei* was the predominant species isolated from 25/58 (43.1%) samples, closely followed by *C. albicans* in 22/58 (38%) samples, Table 2. *C. parapsilosis* was isolated only once from an ICDAS-6 lesion.

**Table 2.** Frequency distribution of isolation of *Candida* species according to caries lesion severity including, mono-, dual- and triple species carriage rates

*Candida* species	ICDAS- 5^#^ Isolation frequency from 43 lesions (n, per cent)	ICDAS- 6^#^ Isolation frequency from 57 lesions (n, per cent)	Total isolation frequency	*P*- value
Mono species carriage				
*C. albicans*	6 (42.9)	5 (11.4)	11	NS
*C. krusei*	3 (21.4)	11 (25)	14	NS
*C. tropicalis*	3 (21.4)	6 (13.6)	9	NS
*C. glabrata*	0	2 (4.5)	2	-
*C. parapsilosis*	0	0	0	-
Dual species carriage				
*C. albicans + C. krusei*	0	2 (4.5)	2	-
*C. tropicalis + C. krusei*	1 (7.1) *	7 (16) *	8	<0.05*
*C. albicans + C. tropicalis*	0	6 (13.6)	6	-
*C. albicans + C. glabrata*	0	1 (2.3)	1	-
*C. tropicalis + C. glabrata*	0	2 (4.5)	2	-
*C. tropicalis + C. parapsilosis*	1 (7.1)	0	1	-
Triple species carriage				
*C. albicans +C tropicalis+ C. glabrata*	0	1 (2.3)	1	-
*C. albicans +C tropicalis+ C. krusei*	0	1 (2.3)	1	-
*TOTAL ISOLATION FREQUENCY*	*14 (100)*	*44 (100)*	*58*	<0.05*

# *Candida* species were isolated from 14 of 43 ICDAS-5 lesions (32.5%) and 44 of 57 ICDAS-6 lesions (77.2%)

*P* values* obtained through Fischer’s exact test and Chi-squared test

**Figure 1.**
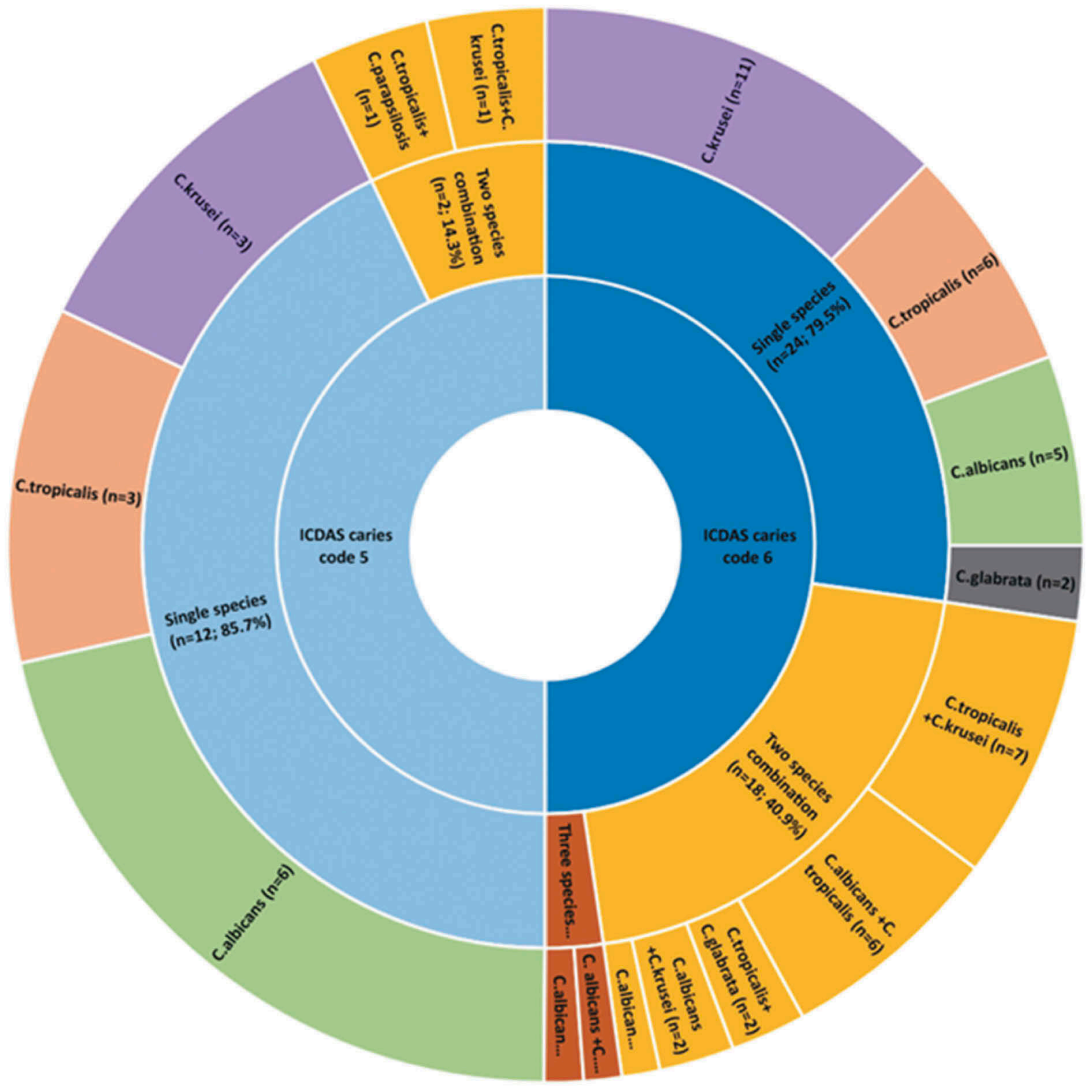
Distribution of *Candida* species according to ICDAS caries lesion severity code 5 (distinct cavity with visible dentin) and caries code 6 (extensive caries lesion involving half/more than half of tooth)

Interestingly, over 50% *of* the deep dentinal lesions (i.e. ICDAS-6) exhibited candidal reservoirs, and in addition, a high propensity for dual/triple species candidal co-colonization. Thus, only 14/43 ICDAS-5 lesions (32.5%) were yeast positive, compared to 44/57 ICDAS-6 lesions (77.2%) (p < 0.05). As for the multi-species presence, we noted a highly significant difference between ICDAS-5 and ICDAS-6 lesions as the deeper lesions harboured more multi-species in comparison to the shallower ICDAS-5 lesions. Hence, in comparison to 20/57 (35.1%) occasions of cohabitation of two/three species of *Candida* in the deeper, ICDAS −6 lesions, only 2/43 (4.7%) demonstrated two-species in ICDAS-5 lesions (p = 0.001; Table 2; Figure 1).

**Figure 2.**
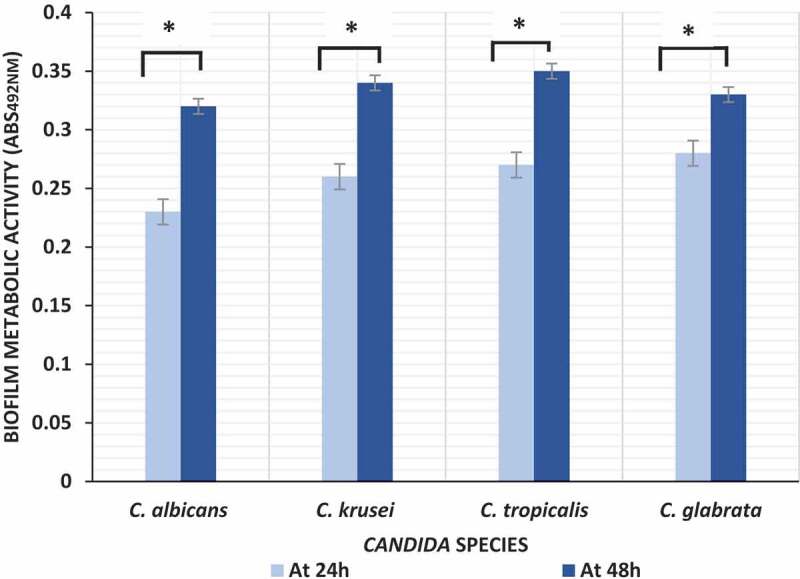
Biofilm formation by *Candida* species (*C. albicans* n = 10; *C. krusei* n = 10; *C. tropicalis* n = 10; *C. glabrata* n = 5) at 24 hand 48 h; *P value < 0.05 (ANOVA)

Biofilms formed by four different *Candida* species were quantified at two different time points using XTT assay for measuring biofilm metabolic activity. No significant differences in inter- or intraspecies biofilm metabolic activity was observed either within or amongst the four *Candida* species, either at 24 or at 48 hr time points (Figure 2).

**Figure 3.**
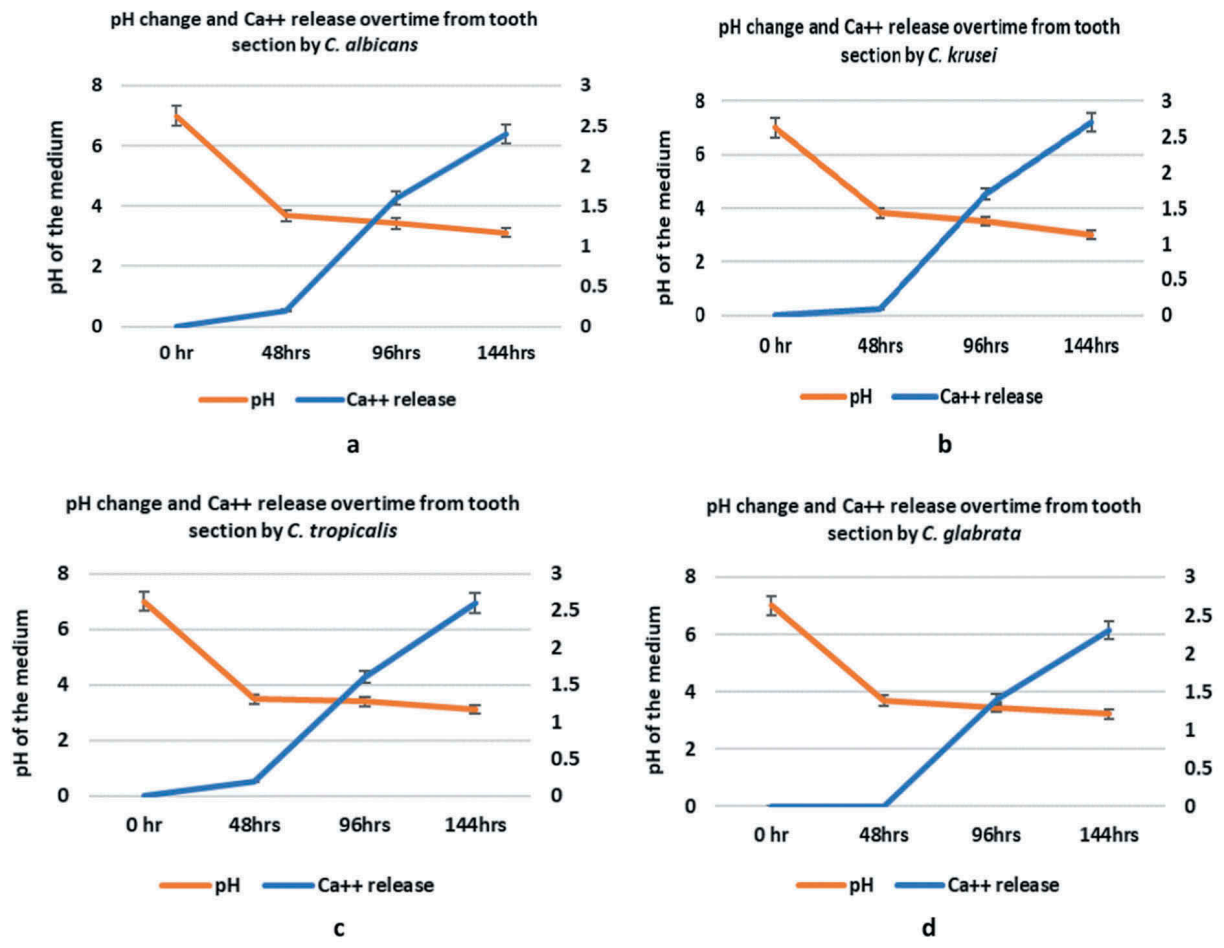
(a–d) Ca++ release assay and pH changes initiated by four different *Candida* species during tooth-demineralization evaluation (**3a**, *C. albicans* n = 10; **3b**, *C. krusei* n = 10; **3c**, *C. tropicalis* n = 10; **3d**, *C. glabrata* n = 5)

All four *Candida* species were uniformly instrumental in provoking a dramatic decline in pH of the SDB medium from pH 7 to below 4.0 over a 48 h period post-incubation, with no significant interspecies difference (p > 0.05; Figure 3(a–d)). After which, at 96 hr and 144 hr time points, a further marginal drop in pH to below 3.5 was noted in all *Candida* suspensions (p > 0.05; Figure 3(a–d)).

All four candidal species demonstrated an equal potency to demineralize the tooth disc substrate, as shown by the Ca++ release into the incubation medium (p > 0.05) – a surrogate indicator of enamel/dentine demineralization (Figure 3(a–d)). As expected, the dissolution of mineralized tooth structure resulting in Ca++ release, was relatively low at 48 h at pH 4, but increased substantially when the pH dropped to (approx.) 3.5 over the remaining incubation period of upto144 h. Though there were no significant inter-species or intraspecies differences in Ca++ release at each time point, there was clearly a significant temporal increase in calcium release from the tooth discs by all four *Candida* spp. between different time points (144 h > 96 h> 48 h) of incubation (p < 0.05).

**Figure 4.**
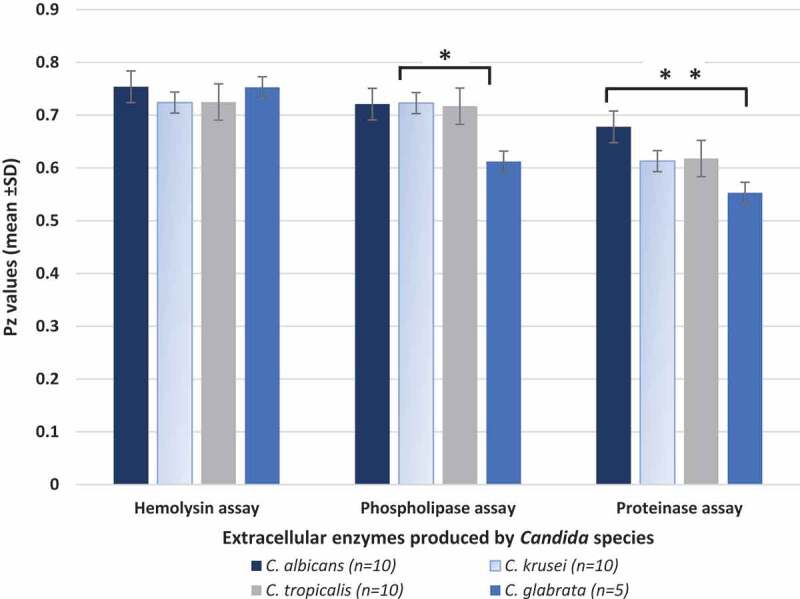
Haemolysin, phospholipase and proteinase production by the four different *Candida* species isolated from deep dentin caries lesions of children with S-ECC

In terms of the hemolysin activity of the evaluated species, we noted that all 35 isolates of *Candida* belonging to the four different species were hemolysin producers. However, no significant inter or intraspecies differences in the hemolysin activity was observed (p > 0.05; Figure 4).

The production of all three hydrolases was designated as **Pz = a/b**, where (a) is the diameter of the colonies and (b) is the diameters of the precipitation zone around the colonies on different media, as described by Price et al. [36]* p < 0.05 phospholipase activity between *C. albicans, C. krusei, C. tropicalis* versus C. glabrata** p = 0.001 -proteinase enzymes activity between *C. albicans* and non-*albicans Candida* species

However, we noted a significant interspecies difference in the mean phospholipase activity levels between *C. albicans, C. krusei, C. tropicalis* versus *C. glabrata* (p < 0.05; Figure 4). *C. albicans* demonstrated the greatest activity with seven of 10 isolates producing phospholipases (data not shown) while only one of five *C. glabrata* isolates was phospholipase positive, and the other two species had intermediate levels of activity (Figure 4). No intra-species differences in phospholipase activity could be discerned (p > 0.05).

Similarly, *C. albicans* isolates exhibited highest levels of proteinase activity compared to the other tested species, with a significant mean difference in activity between *C. albicans* and *C. glabrata* isolates (p < 0.001; Figure 4).

**Figure 5.**
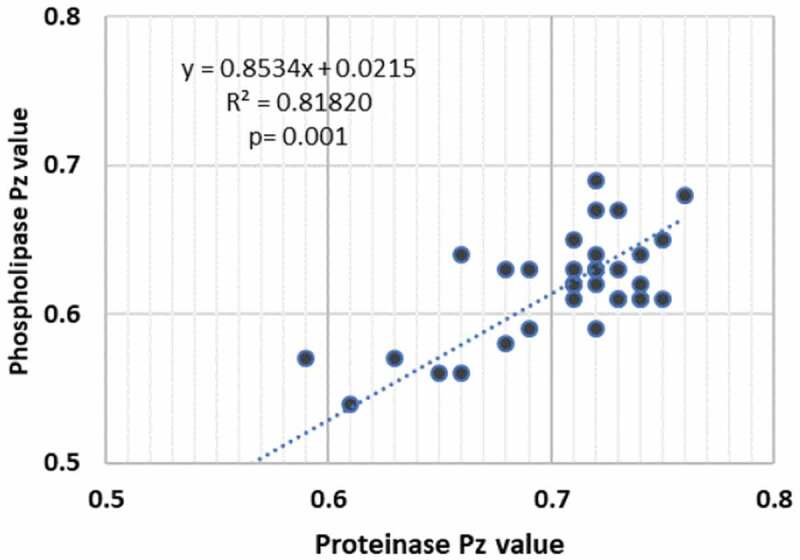
Correlation between the Pz value of proteinase and phospholipase enzyme activity of 29/35 clinical isolates belonging to *C. albicans, C. krusei, C. tropicalis* and *C. glabrata s*pecies (P < 0.001; R^2^ = 0.812)

When we correlated the phospholipase and proteinase activity of a total of 29 *Candida* isolates belonging to the different species, which produced these hydrolases, a highly significant positive correlation between the production of the two extracellular enzymes was noted (r = 0.818; p < 0.001; Figure 5).

Finally, except for a single clinical strain of *C. albicans*, which was DNase positive, none of the other 34 isolates belonging to four different candidal species showed extracellular DNase activity (data not shown).

In conclusion, as regards to the secreted hydrolases, *C. albicans* demonstrated significantly higher protease activity (p < 0.001) as well as phospholipase activity (p < 0.05) relative to the other three non-albicans *Candida* species, while the DNase and hemolysin activity of all four species appeared to be similar (p > 0.05).

## Discussion

Etiopathogenesis of dental caries is intricate and complex. Bacterial and fungal polymicrobial communities in plaque biofilms are now recognized as the prime mover of the caries process [2,39]. A combination of specific-ligand receptor interactions mediating adhesion to biotic/abiotic surfaces, and microbiota-matrix communications via quorum sensing mechanisms, contribute to such biofilm initiation. If these non-specific interactions are arrested during the initial stages of adherence to dental tissues, then biofilm formation can be reversed [17,40]. However, the cariogenic process, once established, particularly in deep cavitated dentinal lesions of S-ECC, is relentless owing to the multiplicity of contributory factors. These include, i) a stagnant niche with accumulated organic debris, ii) a diversely rich bacterial/fungal microbiome, iii) virtual absence of salivary flow and consequent lack of flushing action, and the innate salivary antimicrobial mechanisms, and finally, iv) the predominantly acidic pH milieu in deep carious trenches.

Here, we present novel insight into the fungal microbiota in S-ECC and their virulence attributes. To the best of our understanding, this is the first comprehensive report highlighting fungal existence as mono, dual, and mixed- *C. albicans* and non-*albicans Candida* species, in deep dentinal lesions, as well as their major pathogenic attributes that contribute to enamel demineralization and dentin collagenolysis. Our clinical data, from the largest cohort of S-ECC examined to date unequivocally demonstrate an unexpectedly high prevalence of a spectrum of yeasts in such extensive cavitated lesions (Figure 1). To our surprise we isolated a multitude of common human pathogenic *Candida* species, either singly or in combination, in these caries-active deep-dentin cavities.

Given the abundance of the candidal flora in these lesions, which in itself was an interesting and a novel finding not reported elsewhere, we attempted to evaluate the difference, if any, between the yeast flora in ICDAS-5 and ICDAS 6 lesions. However, we were unable to detect any significant difference or correlation in the severity of the lesions and the prevalence of yeasts. But we noted a startling difference in the multi-species co-habitation, with approximately over one third (35.1%) of the deeper lesions co-colonized as opposed to only a small minority (4.7%) of the shallower cavities. We are unable to attribute a reason for this intriguing finding, but it is tempting to speculate that the deeper lesions with perhaps lower pH and Eh may be conducive to such multi-species co-habitation. Further work, however, need to be done to confirm or refute our contention.

Due to the abundance of the yeast flora and the species variations, we then evaluated their key pathogenic attributes that could possibly contribute to the caries process. In particular, we examined the pathogenic qualities that may lead to enamel/dentine demineralization as well as the dissolution of the organic dentinal matrix components (i.e. collagenolysis). For this purpose, we randomly selected 35 out of a total of 58 yeast isolates belonging to four predominant human candidal pathogens, *C. albicans, C. krusei, C tropicalis*, and *C glabrata* and set out to examine their key, putatively cariogenic virulent attributes. A number of isolates belonging to each species was selected as it is well known that multiple isolates are essential to decipher a specific pathogenic feature of a given species, due to the intraspecies trait differences [41]. Many studies conducted with a single/dual strain belonging to a single species have made this recommendation due to such intraspecies phenotypic variability [41,42].

As mentioned, *Candida* species have an immense capacity to form biofilms on both abiotic and biotic surfaces [39,43]. Yeast biofilm formation is a sequential process beginning with adherence and proliferation of blastospores on the substrate, accumulation of extracellular matrix, and finally, biofilm dispersal. Inter and intraspecies variations on the rate of biofilm formation have been reported amongst *Candida* species although we did not discern such variability between the four tested species [27,44–46]. All four species were uniformly good biofilm-formers over the observation period of 48 hrs. implying their similar capacity to colonize the dentinal niche irrespective of species differences. This is borne out by the fact that both *C. albicans* and *C. krusei*, for instance, were equally predominant colonizers of the deep dentinal plaque, despite the general observation that the former is the superior biofilm former, and the latter lacks the capacity for hyphal development [43].

However, our results on the similar biofilm forming ability of several candidal species could possibly be a reflection of the XTT assay (which evaluates the gross metabolic activity of the biofilm mass) we used, and further work is necessary to confirm or refute this observation. Additionally, biofilm assays in a simulated saliva medium with a dietary carbohydrate supplement such as sucrose should be performed to mimic and reproduce realistic *in vivo* conditions. Also, *in vitro* studies of mixed yeast cultures for biofilm formation and acid production, mirroring the *in vivo* caries niche, should provide additional insights into the cariogenic traits of yeasts.

*Candida* species are supremely adapted to survive and thrive in acidic settings [19,20,24,47]. Some laboratory models have shown the potential of *C. albicans* to rapidly dissolve hydroxyapatite in a low pH milieu [48,49]. Our *in vitro* data on decalcification of tooth samples clearly show that both non-*albicans Candida* and *C. albicans* species released Ca++ from hydroxyapatite in an acidic milieu. Moreover, the whole process was concomitantly accompanied by a drastic reduction in pH from 7.0 to 3.0 over a 24 hr incubation period, plateauing thereafter. It is tempting to speculate that similar mechanisms may operate *in vivo* in deep dentinal cavities of S-ECC, within entombed, stagnant, low pH eco-systems devoid of salivary defenses. Indeed, one of the more powerful virulence traits of *Candida* species is their ability to control the local habitat through their unique metabolic machinery. Being both aciduric and acidogenic, the yeasts, for instance, can metabolize lactate produced by neighboring organisms to short-chain carboxylic acids such as formats and acetates that drive the hard tissue dissolution leading to caries – a mechanism not too dissimilar to that of *mutans*-streptococci [50]. Additionally, Crabtree negative yeasts such as *C. albicans*, can switch between fermentative and respiratory pathways depending on oxygen availability [51] and hence adapt to nutrient and oxygen fluxes extant within the lesion, particularly at its depth [52]. Curiously, we also noted that all four *Candida* species from the S-ECC lesions were uniformly acidogenic and aciduric, a finding that needs further evaluation by comparing strains derived from carious lesions and other non-carious oral co-locales.

Compared to enamel, dentine is made up of a considerable amount (approx. 30%) of organic matter, mainly collagen [53]. Hence, a candidate cariogen needs to possess attributes necessary for proteolytic degradation (collagenolysis) particularly at the advancing front of the deep caries-lesions [52]. Ultrastructural observations on the dentin-demineralization process suggest a two-stage proteolytic process. An initial demineralization of the mineral dentin content [54] exposing the collagen scaffold, and a secondary stage of collagen destruction by the proteolytic enzymes. Additionally, the exposed collagen scaffold appears to serve as a skeleton for further biofilm formation and lesion perpetuation into the pulpal regions [52].

In addition to the ability of *Candida* species to adhere to dentin-collagen [17,55–57], they also possess proteinases active in acid media. Wu and Samaranayake [58] have demonstrated in early studies, the intraspecies variation in proteinase production of *Candida* species in *in vitro* salivary cultures as well as in artificial media. They noted that *C albicans* is a superior proteinase producer than *C. tropicalis* and *C. parapsilosis* [58]. Our results tend to confirm the latter hierarchy of virulence in *Candida* spp., as we too noted that *C. albicans* was the predominant protease producer amongst the all four tested species. Similarly, it is well recognized that *C. albicans* is the foremost phospholipase producer in comparison to its counterparts [59], and this was borne out by the current data where 70% of the *C. albicans* strains produced this potent enzyme.

Another interesting finding was the positive association between the phospholipase and protease production amongst all the strains that produced both these enzymes. There is one study that has noted a positive association between these two hydrolases [60], while others have been unable to show such an association. This implies that caries- associated candidal flora may have a relatively strong armamentarium, which facilitates collagenolysis. In general, though, the inter- and intra-species phenotypic variations reported above amongst the caries-associated *Candida* species implies that our findings are not too dissimilar to those of others who compared the virulence attributes of oral, vaginal, urinary and various other body sites [35,61].

Taken together, our findings imply that candidal flora play a critical role in S-ECC, and a deeper carious niche is conducive to multi-species habitation in comparison to more superficial lesions. Furthermore, it appears that, in addition to their prodigious and well recognized acidogenic and aciduric potential, the collagenolytic virulence traits of candidal species could be an additional contributing factor for the now apparent major role they play in S-ECC. Further, similar work in different child cohorts, in various geographic locales, is essential to confirm our findings, most of which are reported here for the first time.
